# Functional and cosmetic outcome after Septorhinoplasty - A retrospective study

**DOI:** 10.6026/973206300220599

**Published:** 2026-01-31

**Authors:** Ajazul Haq, Sandeep Singh, Preeti Sharma, Deep Jyoti, Rupali Sharma, Pryianka Wahi, Kamal Kishore

**Affiliations:** 1 Department of ENT & HNS, Government Medical College, Jammu and Kashmir, India

**Keywords:** Septorhinoplasty, functional, cosmetic outcomes

## Abstract

The goal of surgical septorhinoplasty is gives the nose an appearance that complements the surrounding facial features and permits an unhindered airway. Therefore, it is of interest to evaluate functional and cosmetic outcome after septorhinoplasty using Rhinoplasty Outcome Evaluation (ROE), peak nasal inspiratory flow (PNIF). Patient were satisfied with their post ROE in 10 (26.31%) males and 14 (36.84%) females. Satisfaction regarding post-operative PNIF was observed in 10(26.31%) males and 12 (31.57%) females. After a septorhinoplasty, nasal blockage and outward appearance significantly improved and the ROE evaluation and the PNIF monitor make it simple to gauge how satisfied patients are after a septorhinoplasty.

## Background:

In terms of both physiological and anatomical characteristics, the nose along with the sinus cavities that are connected to nose at the start of the upper airway constitute a complex unit [1-2]. Their primary roles include regulating air temperature, humidifying and filtration of inspired air, and protecting the lower part of the airway. It is mediated through an immune reaction against pollutants, allergens, and other kinds of particles [3]. A person's appearance and expression are greatly influenced by their nose [2]. A surgical treatment called septorhinoplasty is done to enhance the nose's look and functionality [2]. Since ancient times, septorhinoplasty has been carried out. Edwin Smith Papyrus, a reproduction of an Egyptian medical literature of ancient times has the first recorded mention of plastic nose repair [4, 5]. The Ayurvedic healer Sushruta (c. 800 BC) practiced rhinoplasty methods in ancient India. He mentions nose repair in his Sushruta Samhita. In order to create the rhinoplasty procedure, Sushruta had to remove skin that covered forehead, which was supplied with blood by the angular artery [4, 5-6]. Afterwards on, Gaspare Tagliacozzi was able to effectively remodel several patients' noses using the arm flap technique [3, 4-5]. In the realm of rhinoplasty procedures, particularly reduction rhinoplasty, Jacques Joseph being considered a leader. Advanced aesthetic surgery is credited to Carl Ferdinand von Gräfe. Because he was dissatisfied with the donor site scarred morbidity caused by the forehead rhinoplasty, von Gräfe preferred the upper arm flap surgery [3, 4-5]. Nocini *et al.* improved and popularized the transition from the closed to the open rhinoplasty procedure, which Goodman started [7, 8-9]. The surgical objective is to enhance the patient's look. It is done by providing the nose a natural look that blends in with the adjacent facial characteristics. It also allows for an unimpeded airway [10, 11-12].

Patient outcomes are of importance to all surgeons. Instead of concentrating only on conventional clinical measurements of treatment effectiveness, outcome research examines these outcomes. It is carried out by using a variety of patient centric metrics including contentment, performance, and overall quality of life [13, 14- 15]. Septorhinoplasty is deemed successful if the patient is satisfied and their quality of life improves, as is the case with any cosmetic procedure [16, 17- 18]. According to the literature, rhinoplasty patients are less satisfied with the achieved results in comparison with those who underwent other facial plastic surgery procedures [19, 20-21]. Due to its aesthetic nature, having a great impact on the self-image and self-esteem of the patient, the assessment of the final rhinoplasty results should be made from the patients' viewpoint [22, 23-24]. Imposing criteria that determine aesthetics is not an easy task. For this reason, numerous questionnaires are available to evaluate quality-of-life change after the procedure [25]. A simple questionnaire called the ROE was created to gauge how satisfied patients were and the end result of rhinoplasty. There are six questions in all, two for each of the three aspects of patient satisfaction-social, emotional, and physical. Since its inception, it has gained popularity as an assessment technique and has been incorporated into other languages [3]. There are several objective techniques to determine passage of air from nose, nasal resistance to airway obstruction nasal passageway measurements. Although such methods yield useful data, they are not as feasible for healthcare professionals. It is due to their time commitment, high equipment costs, and need for skilled personnel. The evaluation of PNIF is quick, inexpensive, harmless, and capable of giving an unbiased assessment of nasal airflow in immediate time. Therefore, it is of interest to evaluate functional and cosmetic outcome after septorhinoplasty using ROE and PNIF and to evaluate advantages of these assessment methods to gauge how satisfied patients are after a Septorhinoplasty.

## Materials and Methodology:

A retrospective study was carried out to quantify the surge in nasal airflow among ENT department patients who underwent Septorhinoplasty and to identify benefits in both functional as well as cosmetic characteristics.

## Criteria for inclusion:

[1] Every patient who was older than 18.

[2] Every primary case.

## Criteria for exclusion:

[1] Individuals who have cleft lip with or without cleft palate.

[2] Individuals suffering from Lefort fractures.

[3] Individuals who already have a psychiatric diagnosis.

## Method of data collection:

Medical records of every patient who arrived at the ENT OPD with an exterior deformity and nasal obstruction during period of October 2024 to May 2025 were obtained. Reports of nasal endoscopy were collected that was done for diagnostic purposes. Details of 38 patients who underwent Septorhinoplasty were obtained. Pre-operative data based on The PNIF meter and the ROE Questionnaire was gathered. Findings of patients reassessed three months after surgery, using the same scales were obtained. Data from both of these circumstances were then compared. Pictures of patient both before and after surgery were obtained. All patients received a single injection of ceftriaxone prior to surgery. A head ring is used to elevate the patient's head to approximately 75° while they are in a supine position. General anesthesia was used during the surgery. Adrenaline (1:80,000) along with 1% lignocaine was injected into the nose. For proper anterior nasal packing assistance, patients wore an external splint using plaster of Paris (POP). All patients received intravenous antibiotics and painkillers (paracetamol) after surgery. On the first post-operative day, the anterior nasal pack was taken away. On the first post-operative day, the patients were released. On the first OPD visit one week following surgery, POP had been removed. The external stitches were taken out under the same conditions. Every three months, patients were monitored. At that moment, pictures were taken. Data from the ROE questionnaire and PNIF meter was gathered, and nasal breathing was evaluated.

## Statistical analysis:

Statistical analysis was conducted using the paired t-test to evaluate differences between pre- and post-intervention measurements. The corresponding p-values were computed to determine the level of significance. A threshold of p < 0.05 was considered statistically significant, indicating a less than 5% probability that the observed differences occurred by chance.

## Results:

Table 1 describes the age distribution of study participants reveals a predominant concentration in younger age groups. Out of a total sample size of 38 individuals, 42.10% were between 11-20 years of age, comprising the largest segment. This was followed by 36.84% in the 21-30 age brackets, reflecting strong representation among young adults. Middle-aged individuals accounted for fewer entries, with 15.78% between 31-40 years and only 5.26% in the 41-50 range. This skewed distribution suggests the study population is largely composed of younger individuals, which may influence outcomes related to age-dependent variables and should be considered in the interpretation of the results. Table 2 describes the study comprised 38 participants, with a gender distribution showing a modest female predominance. A total of 42.10% (n = 16) were male, while 57.90% (n = 22) were female. This balanced representation supports meaningful gender-based analysis, while highlighting the slight overrepresentation of females in the sample. Table 3 describes the occupational distribution of the study participants indicates that the majority were children or students, accounting for 52.63% (n = 20) of the total sample. Homemakers comprised 21.05% (n = 8), suggesting a significant representation of non-working adults engaged in household responsibilities. Professionals formed 15.78% (n = 6), while skilled workers represented 10.52% (n = 4).

Table 4 describes the analysis of Rhinoplasty Outcome Evaluation (ROE) scores reveals a highly significant improvement in patient satisfaction following the surgical procedure. The mean preoperative ROE score was 30.81 (SD = 16.36), indicating relatively low baseline satisfaction, while the postoperative score rose markedly to 88.43 (SD = 9.86), reflecting substantial enhancement in subjective outcomes. The standard error of the mean (SE) for pre- and post-operative scores were 3.62 and 2.12, respectively, suggesting greater precision in the post-surgical assessments. The standard error of the mean (SE) for pre- and post- operative scores were 3.62 and 2.12, respectively, suggesting greater precision in the post-surgical assessments. A paired t-test was employed to assess the statistical significance of these changes, yielding a t-value of 16.25 with 189 degrees of freedom. The resulting two-tailed p-value was less than 0.0001, confirming that the observed difference is statistically significant. The 95% confidence interval for the difference in scores ranged from -67.62 to -50.65, further reinforcing the reliability and magnitude of the improvement. These findings strongly support the effectiveness of rhinoplasty in enhancing patient-reported outcomes and satisfaction. Table 5 describes the significant improvement in Peak Nasal Inspiratory Flow (PNIF) was observed following surgery. The mean preoperative PNIF value was 77.69 (SD = 42.48), which increased to 126.00 (SD = 51.33) postoperatively, indicating enhanced nasal airflow. The standard error of the mean was 10.50 before surgery and 12.63 after, reflecting acceptable measurement precision in both conditions. Statistical analysis using the paired t-test revealed a t-value of 7.09 with 19 degrees of freedom and a p-value of <0.0001, suggesting that the improvement in PNIF was highly statistically significant. The 95% confidence interval for the difference ranged from -63.11 to -34.97, confirming that the postoperative enhancement was both reliable and clinically meaningful. Table 6 explains about patient satisfaction. The patient were satisfied with their post ROE in 10 (26.31%) males and 14 (36.84%) females. Satisfaction regarding post-operative PNIF was observed in 10(26.31%) males and 12 (31.57%) females. Patients satisfied with both post-op ROE and PNIF results were 8 (21.05%) in both males and females (Table 6).

## Discussion:

One unique face feature of significant aesthetic value is the nose. Being the primary hallmark of the face, its symmetry, size, and separation from other anatomical features all have an impact on how beautiful the face looks. It is essential to each person's unique identity. Among other things, the nose's regularity and spatial relationships greatly influence the general equilibrium and beauty of the face. Perhaps the most frequent operations carried out in the medical specialties of plastic surgery and otolaryngology is rhinoplasty. Obstructive nasal passages and attractiveness are the primary indications [1]. One of rhinology's main objectives has been to measure the nose's breathing and ventilation. These evaluations are not directly associated with patient-reported nasal breathing. However they do generally correspond with other objective techniques used in nasal airway evaluation. Nevertheless, low PNIF values make it difficult to perceive appropriate nasal airflow. PNIF assessment may also be helpful in determining the best course of action for patients who complain of nasal airway blockage. The goal of septorhinoplasty is to improve the patient's external disfigurement and nasal blockage. We included 38 individuals who had septorhinoplasty in our study. Of the patients, 42.1% were between the ages of eleven and twenty. Just 5.3 percent of the individuals receiving treatment were between the ages of 41 and 50. This could be because younger people are more concerned about appearance, and as they get older, nasal blockage can lower their quality of life. The study population consisted of 16 (42.10%) men and 22 (57.90%) women, indicating that women are generally concerned with both functional and esthetic issues. A research found an analogous distribution across sexes. Sixty-two percent of their 88 patients were female [13]. Our research's average patient age was 26.10 years, whereas other study's had been 37.6 years. In our research, pre-operatively, mean ROE scores was30.81±16.36 and postoperatively mean ROE scores was 88.43±9.86. After surgery, none of the participants in our research experienced a decline in their scores. 12 patients (63.16%) had a post-op value of >85, which is regarded as outstanding. of which seven were female and five were male. Following septorhinoplasty, ROE scores significantly improve (p<0.0001). This suggests that nasal blockage and cosmetic issues are resolved via septorhinoplasty. A quick, easy, and useful subjective evaluation tool for patients having septoplasty is the ROE questionnaire. Similar outcomes were obtained in a clinical trial including 19 patients, with a prior to operation ROE rating of 24.6±11.3 that increased to 76.1±19.5 after surgery. A study found that the ROE questionnaire significantly improved the standard of life following rhinoplasty [10]. An extended study with sixty-nine patients following septorhinoplasty was carried out and found that the ROE questionnaire yielded a mean result of 73.25 percent [15]. Another study evaluated the wellbeing of life in relation to nasal functionality and attractiveness following rhinoplasty, and they discovered that ROE values improved after surgery (p <0.01) [16]. According to the ROE questionnaire, 87.5% of patients who had a sphenorhinoplasty had excellent post-operative outcomes in a research [17]. Another study found that the average variance between prior to operation and after the procedure ROE values was 54.26±18.85, indicating a statistically noteworthy with betterment in ROE scores after sphenorhinoplasty [2]. Mean PNIF values before surgery was 77.69±42.48 while PNIF values after surgery was 126±51.33. The improvement in PNIF after Septorhinoplasty was significant statistically (p<0.0001). After surgery, 22 patients-10 of whom were male and 12 of whom were female-had normal values (> 120 L/min). Four individuals were unable to complete the test prior to surgery. It can be the result of a significant nasal blockage that made the test technically challenging to administer. For a male 22-year-old, the maximum before surgery PNIF number is 180. The greatest recorded post-op value was 260, which was his post-op value. For a patient having a pre-op value of 0, the minimum following surgery PNIF value is 50. The patient's pre-op value was 80, but their post-op value was 55. A decreased number after surgery is likely due to the patient's infection of the upper respiratory system during the period of follow-up. In a research by Timperley *et al.* the average PNIF value before surgery was 86.5 L/min, while the average PNIF value after surgery was 123 L/min, indicating a substantial recovery (p < 0.05). In a research by Timperley *et al.* the prior to surgery average PNIF was 101±35 L/min, which enhanced to 143±44 L/min [18]. Following septorhinoplasty using a PNIF meter, 136 participants in an American research showed statistically significant improvements in their nasal airways. L/min raised from 82.3 upto 108.4 [19]. According to Jadczak *et al.* study, which involved 78 participants, PNIF is a dependable technique for identifying fluctuations in nasal functionality because it is simple to use, affordable, and repeatable [20].

## Advancement to knowledge:

Newer assessment methods like ROE evaluation and the PNIF monitor were assessed in this study. It was found that ROE evaluation and the PNIF monitor make it simple to gauge how satisfied patients are after a septorhinoplasty. This will help in enhancing knowledge of clinicians regarding assessment of septorhinoplasty. This study evaluated clinical outcomes of septorhinoplasty using these methods and found significant results.

## Study limitations:

Our study only included 38 participants, which is a fairly small number. It is most likely the outcome of patients' ignorance of the procedure and its aftermath.

## Conclusion:

Patients who get a septorhinoplasty see significant enhancements in both their functional and esthetic features. After a septorhinoplasty, nasal blockage and outward appearance significantly improves. The ROE evaluation and the PNIF monitor make it simple to gauge how satisfied patients are after a septorhinoplasty. Newer assessment methods like ROE evaluation and the PNIF monitor were assessed in this study. It was found that ROE evaluation and the PNIF monitor make it simple to gauge how satisfied patients are after a septorhinoplasty.

## Figures and Tables

**Table 1:** Details about age groups of patients

	**11-20**	**21-30**	**31-40**	**41-50**
Frequency	16	14	6	2
Percentage	42.1	36.84	15.78	5.26

**Table 2:** Details about details gender of patient

**Gender**	**Number**	**Percentage**
Male	16	42.1
Female	22	57.9

**Table 3:** Details about profession

**Occupation**	**Professional**	**Skilled worker**	**Home maker**	**Children/student**
Number of patients	6	4	8	20
Percentage (%)	15.78	10.52	21.05	52.63

**Table 4:** Paired t test outcomes of ROE pre surgery and post-surgery

		**ROE preop**	**ROE post-op**
Mean		30.81	88.43
SD		16.36	9.86
SE mean		3.62	2.12
95% Confidence interval of the difference	Lowest	-67.62	
	Highest	-50.65	
t	16.2492		
df	189		
Sig (2 tailed)	<0.0001		

**Table 5:** Paired t test results of PNIF before and after surgery

		**PNIFbefore surgery**	**PNIFafter surgery**
Mean		77.69	126
SD		42.48	51.33
SE mean		10.5	12.63
95% Confidence interval of the difference	Lowest	-63.11	
	Highest	-34.97	
t		7.0923	
df		19	
Sig (2 tailed)		<0.0001	

**Table 6:** Satisfaction following surgery

	**Males**	**Females**
ROE post-op	10 (26.31%)	14 (36.84%)
PNIF post-op	10 (26.31%)	12 (31.57%)
Both post-op ROE and PNIF	8 (21.05%)	8 (21.05%)

## References

[1] Patel PN (2023). Aesthetic Plast Surg..

[2] Sasindran V (2020). Indian J Otolaryngol Head Neck Surg..

[3] Xavier R (2024). Facial Plast Surg..

[4] Wells MW (2023). Aesthetic Plast Surg..

[5] Villegas-Alzate F (2024). Aesthetic Plast Surg..

[6] Mazzola IC, Mazzola RF (2014). Facial Plast Surg..

[7] Nocini R (2022). Ann Maxillofac Surg..

[8] Rot P (2024). J Clin Med..

[9] Plath M (2021). Acta Otorhinolaryngol Ital..

[10] Wähmann MS (2018). Aesthetic Plast Surg..

[11] Alsarraf R (2000). Aesthet Plast Surg..

[12] Barone M (2017). Eur Arch Otorhinolaryngol..

[13] Strazdins E (2018). JAMA Facial Plast Surg..

[14] Marangi GF (2024). Aesthetic Plast Surg..

[15] Stergiou G (2022). Aesthetic Plast Surg..

[16] Fuller JC (2018). JAMA Facial Plast Surg..

[17] Chen K, Zhou L (2024). Aesthetic Plast Surg..

[18] Timperley D (2010). Int Rhinol Soc..

[19] Tabrizi AG (2024). World J Plast Surg..

[20] Jadczak M (2024). J Clin Med..

[21] Başer E (2016). J Craniofac Surg..

[22] Yadav R (2025). Cureus..

[23] Papadopulos NA (2021). Facial Plast Surg..

[24] Yilmaz GO (2025). Actet ala Otolaryngol..

[25] Barone M (2017). Eur Arch Otorhinolaryngol..

